# Simple and complex cells revisited: toward a selectivity-invariance model of object recognition

**DOI:** 10.3389/fncom.2023.1282828

**Published:** 2023-10-13

**Authors:** Xin Li, Shuo Wang

**Affiliations:** ^1^Department of Computer Science, University at Albany, Albany, NY, United States; ^2^Department of Radiology, Washington University at St. Louis, St. Louis, MO, United States

**Keywords:** simple and complex cells, selectivity computation, invariance computation, object recognition, cortically local subspace untangling

## Abstract

This paper presents a theoretical perspective on modeling ventral stream processing by revisiting the computational abstraction of simple and complex cells. In parallel to David Marr's vision theory, we organize the new perspective into three levels. At the computational level, we abstract simple and complex cells into space partitioning and composition in a topological space based on the redundancy exploitation hypothesis of Horace Barlow. At the algorithmic level, we present a hierarchical extension of sparse coding by exploiting the manifold constraint in high-dimensional space (i.e., the blessing of dimensionality). The resulting over-parameterized models for object recognition differ from existing hierarchical models by disentangling the objectives of selectivity and invariance computation. It is possible to interpret our hierarchical construction as a computational implementation of cortically local subspace untangling for object recognition and face representation, which are closely related to exemplar-based and axis-based coding in the medial temporal lobe. At the implementation level, we briefly discuss two possible implementations based on asymmetric sparse autoencoders and divergent spiking neural networks.

## 1. Introduction

How do we learn to see in the first 6 months after birth? To answer this question, David Hubel and Torsten Wiesel conducted pioneering experiments in the 1950s, leading to the discovery of simple and complex cells (Hubel, 1995). Inspired by their discovery, David Marr developed a theory of the neocortex (Marr and Thach, 1991) in 1970 and a theory of the hippocampus (Marr et al., 1991) in 1971. His computational investigation of vision (Marr, 2010) was published in 1982 after his death. The construction of neocognitron (Fukushima, 1984) and connectionist models (Rumelhart et al., 1985) in the 1980s represented the continuing effort to construct biologically plausible computational models for object recognition. Wavelet theory (Mallat, 1989) and sparse coding (Olshausen and Field, 1996) in the 1990s further supplied mathematical formulations of multi-resolution analysis for scale-invariant representation of images. Rapid advances in deep learning (Goodfellow et al., 2016), especially the class of over-parameterized models (Arora et al., 2018; Allen-Zhu et al., 2019) have expedited both the theory and practice of learning-based visual information processing.

Despite the remarkable progress of today, the gap between biological and artificial vision remains significant in the following aspects. First, the network architecture of the convolutional neural network (CNN) is characterized by the pooling of layers, which reduces the dimensionality of the input data. This is in sharp contrast to the increase in the number of neurons and synapses as we move from the lower layer (e.g., V1) to the higher layer (e.g., V4). This anatomical finding inspired Barlow (2001) to revise his redundancy reduction hypothesis into the redundancy exploitation hypothesis in 2001. Second, CNN still lacks generalizability, which extends far beyond our direct experience (Greff et al., 2020). This shortcoming has been conceived to be related to the binding problem, closely related to consciousness (Treisman, 1996), which has been extensively studied in psychology. A compositional approach to AI is fundamentally important in achieving human-level generalization. Finally, it remains a mystery how the human brain can manage to achieve the objectives of learning and memory with more than 100–1,000 trillion synapses but a power budget of <20W.

The motivation behind this paper is 2-fold. On the one hand, both the human brain and CNN are characterized by the ability to optimize an astronomical number of synaptic weights. The class of over-parameterized models (Arora et al., 2018; Allen-Zhu et al., 2019) has shown some counterintuitive properties, such as double descent (Nakkiran et al., 2021). Analytical tools such as neural tangent kernel (NTK) (Jacot et al., 2018) offer an approach to understanding over-parameterization, but, as with all kernel methods, the assumption with the mathematical structure of the Hilbert space is never justified (Poggio and Girosi, 1990). We seek to understand over-parameterization under the framework of exploring the tradeoff between selectivity and invariance (a.k.a. tolerance) computation instead (DiCarlo et al., 2012). Since nature does not have foresight, it recycles old solutions (e.g., cognitive maps Whittington et al., 2022b) to solve new problems (e.g., object recognition). On the other hand, an evolutionary perspective on biological and artificial neural networks (Hasson et al., 2020) offers a direct-fit approach to understanding biological neural networks. Such a deceivingly simple model, when combined with over-parameterized optimization, offers an appealing solution to increase the generalization (predictive) power without explicitly modeling the unknown generative structure underlying sensory inputs. Along this line of reasoning, neural population geometry offers a “manifold untangling” (DiCarlo et al., 2012; Chung and Abbott, 2021; Li and Wang, 2023) perspective toward understanding the mechanism of ventral stream processing.

In this paper, we follow Marr (2010) vision and study the problem of object recognition at three different levels. At the computational level, we construct an over-parameterized selectivity-invariance model for object recognition. Inspired by the contrast between the mechanisms of selectivity and invariance/tolerance in ventral stream processing (DiCarlo et al., 2012), we make a new proposal to abstract simple and complex cells into space partitioning and composition in a topological space. Unlike existing hierarchical models for object recognition, we advocate a direct-fit approach on the topological manifold that is both biologically plausible and computationally efficient. At the algorithmic level, we connect our construction with a hierarchical generalization of the sparse coding strategy, making it suitable for disentangling object identity from environmental uncertainty factors (e.g., pose, scale, position, and clutter). A new insight brought about by our hierarchical generalization of sparse coding is the dynamic programming (DP)-like solution to approximate nearest neighbor (ANN) for object recognition. Finally, at the implementational level, we briefly discuss two possible implementations based on asymmetric sparse autoencoder (Ng et al., 2011) and divergent spiking neural networks (SNN) (Izhikevich, 2006), respectively. Unlike the original construction, asymmetric sparse autoencoder (Ng et al., 2011) exploits the blessing of dimensionality by formulating mask-task learning in the latent space. Similarly, a divergent SNN attempts to simulate the parallel and sequential binding mechanism of polychronization neural groups (PNGs), which solves the problem of combinatorial arrangement (Damasio, 1989).

## 2. Computational level: over-parameterized selectivity-invariance model for object recognition

In neuroscience, the principle of hierarchical organization can be roughly stated as follows. The nested structure of the physical world is mirrored by the hierarchical organization of the neocortex. This principle was partially inspired by Mountcastle (1957) universal cortical processing algorithm. If Mountcastle were correct, the “simple discovery” made by Hubel and Wiesel might have deeper implications in the mechanism of visual processing beyond V1. The solution, as manifested by an infant's development of the visual cortex (primarily for the ventral stream for object vision) during the first 6 months after birth (Mumford, 2002), lies in a novel construction of an over-parameterized selectivity-invariance model based on the hierarchical organization of simple and complex cells. We advocate the following principle as the key to unlocking the secret of ventral stream processing.

**Principle of dichotomy learning**

*Based on the principle of Hebbian learning, the population of neurons participates in either selectivity or invariance computation. The former generates new patterns/objects about the external environment and is expansive in nature; the latter clusters perceptually equivalent patterns about internal representations (Rumelhart et al.*, *1985**) [a.k.a. mental models (Sterman*, *1994**)] and is compressive in nature*.

Simple and complex cells discovered by Hubel and Wiesel are two concrete examples of selectivity and invariance computation. In this paper, we revisit the problem of modeling simple and complex cells and propose a generalized abstraction/strategy from the perspective of dichotomy learning.

### 2.1. Computational abstraction of simple and complex cells

A contemporary view of ventral stream processing is that the visual ventral pathway gradually untangles information about objects through nonlinear selectivity and invariance computation (DiCarlo et al., 2012). From V1 and V2 to V4 and inferior temporal (IT), each area in the visual cortex plays the role of conveying a neural population-based re-representation of visually presented images. Despite the extensive study of V1 (Olshausen and Field, 2005), our understanding of the processing of the ventral stream in higher stages (V4 and IT) remains poor. It is unclear how the ventral stream produces an IT population representation that can achieve the objective of robust and invariant object recognition. It has been speculated that the problem of manifold untangling is solved hierarchically by a canonical meta-job description of each local cortical subpopulation. This has been called “cortically local subspace untangling” (DiCarlo et al., 2012), which aims to best untangle the object manifold locally (i.e., within the data subspace provided by the input afferents).

Existing bottom-up encoding models are often inspired by Hubel and Wiesel's ground-breaking discoveries of simple and complex cells in 1962. Simple cells implement AND-like operations on lateral geniculate nucleus (LGN) inputs to promote the selectivity of visual stimuli (e.g., directional selectivity). Complex cells implement OR-like operations to achieve a form of invariance (e.g., translational invariance). The combination of simple and complex cells has been the basis of many vision models including neocognitron (Fukushima, 1984), convolutional neural network (CNN) (LeCun et al., 1995), and HMAX (Riesenhuber and Poggio, 1999). However, these existing models share a common convergent architecture due to the max-pooling operator and the associated misconception about the curse of dimensionality. None of them is consistent with Barlow (2001) redundancy exploitation hypothesis based on the observation that the number of neurons does not decrease but increases dramatically at higher stages. Why do we need more neurons in IT than in V2 or V4?

The purpose of this work is to shed some new insight from the perspective of selectivity and invariance computation. The objective of constructing a selectivity-invariance model is to facilitate the task of manifold untangling (DiCarlo et al., 2012)—i.e., how does a population of neurons simultaneously distinguish different stimuli (selectivity) and recognize the same class (invariance)? Our intuition is that nature has discovered an elegant solution to the manifold untangling problem—i.e., the resulting population-based representation of IT single neurons is so powerful that it simultaneously conveys explicit information about all tangled factors including identity, position, size, pose, context, and so on. To understand how a selectivity-invariance model facilitates manifold untangling, we revisit the computational abstraction of simple and complex cells by articulating specific objectives of selectivity-invariance computation. To facilitate our computational abstraction, we briefly review the definitions of subspace and product topology (Lee et al., 2009) below.

**Subspace topology**: Let **X** be a topological space and let **S**⊆**X** be any subset. Then $T_{S}=\left\{\text{U}\subseteq \text{S}:\text{U}=\text{S}\cap \text{V}\;for\;some\;open\;subset\;\text{V}\subseteq \text{X}\right\}$ is the subspace topology.

**Product topology**: Suppose that **X**_1_, …, **X**_*n*_ are topological spaces. In its Cartesian product **X**_1_×… × **X**_*n*_, the product topology is generated on the following basis: $B=\left\{{\text{U}}_{1}\times ...\times {\text{U}}_{n}:{\text{U}}_{i}isanopensubsetof\;{\text{X}}_{i},i=1,...,n\right\}$.

**Simple cells** play the role of vector quantization or space partitioning, which generalizes the conventional wisdom of direction selectivity (Hubel, 1995) and can be abstracted as subspace topology (Kelley, 2017). A good example to understand space partitioning is the *k*-dimensional tree (*k*-d tree) (Bentley, 1975), a space partitioning data structure to organize points in a *k*-dimensional space. The *k*-d tree structure directly fits the data, using hyperplanes to recursively partition the *k*-dimensional space. A simple variant of the *k*-d tree, named the random projection tree (Dasgupta and Freund, 2008), is capable of automatically adapting to the low-dimensional structure of the data without explicit manifold learning. In summary, simple cells distinguish different classes of stimuli by selective firing; from this perspective, even grandmother cells (a.k.a. “Jennifer Aniston neuron”) that only respond to a specific class of stimulus (Quiroga et al., 2005) can be categorized as simple cells regardless of their affiliation with the higher-level cortical regions.

**Complex cells** play the role of product quantization (Jegou et al., 2010) or space composition, which generalizes existing grouping operations and can be abstracted by product topology (Kelley, 2017). Note that our intuition is consistent with the max/sum-pooling operations in the HMAX model (Riesenhuber and Poggio, 1999) because the objective of complex cells is to achieve spatial invariance within an increased field of view. The difference lies in the way of abstraction—from simple to complex cells, we ask what will be its dual operation of space partitioning. Along this line of reasoning, if simple cells are responsible for linear separability without the change of dimensionality; complex cells must increase the dimensionality for handling more sophisticated objects with a larger field of view (up to a certain class of geometric transformations). In summary, complex cells are responsible for invariant recognition—they achieve this objective by explicitly encoding identity-preserving transformations (DiCarlo et al., 2012).

**Figure 1:**
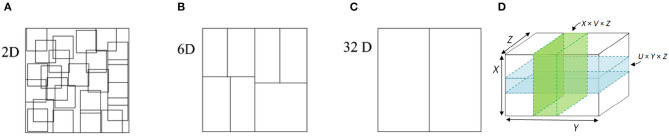
Abstraction of simple/complex cells into space partitioning or subspace topology **(A–C)** and space composition or product topology **(D)**. Note that space partitioning and composition promote selectivity and invariance computation, respectively.

To illustrate the difference between space partitioning and space composition, Figure 1 contains several examples in varying dimensions. Figures 1A–C show how a 2D/6D/32D space can be partitioned into overlapping and nonoverlapping subspaces. Such subspace topology promotes the selectivity computation by simple cells in the space of sensory stimuli. Figure 1D illustrates the composition of subspaces (i.e., the pair of green/vertical or blue/horizontal planes) into a product topology (the higher-dimensional space *R*^3^). Such product topology is useful for abstracting invariance computation of complex cells.

### 2.2. Selectivity and invariance computation along the hierarchy

**Hierarchical model construction** combines layers of simple and complex cells similar to HMAX (Riesenhuber and Poggio, 1999) or neocognitron (Fukushima, 1984), but with an important distinction. The network architecture of our model is not convergent, but *divergent*—one way of generalizing is to still use pooling layers, but we will consider many parallel pooling layers simultaneously. This divergent architecture is inspired by the hypothesis of redundancy exploitation (Barlow, 2001) advocated by H. Barlow, since the number of neurons increases significantly as we move to the higher level of the visual cortex (e.g., from V4 to IT). In addition to the argument with combinatorial coding (Damasio, 1989), we note that achieving spatial invariance by max-pooling loses resolution. To compensate for this sacrifice, context aggregation by dilated convolutions (Yu and Koltun, 2015) has been developed for semantic segmentation. Mathematically, a dilated convolution is equivalent to a convolution without the follow-up max-pooling operation. Alternatively, we can still use max-pooling, but consider the generalization of dilated convolutions to multiple deformable convolutions in parallel. To achieve invariance of object recognition to geometric transformations, we need to construct identity-preserving transformations from local to global along the hierarchy.

To construct a hierarchical model, we note that the combination of space partitioning (by simple cells) and space composition (by complex cells) can be recursively applied to achieve the objective selectivity-invariant computation in an increasingly higher-dimensional space. This recursion is conceptually similar to multi-resolution analysis (Mallat, 1989) but operates in a data-adaptive manner. At each level, the concatenation of simple and complex cells will map visual stimuli to a sequence of invariant representations with increasing dimensionality, selectively requiring more and more polychronization neural group (PNG) (Eguchi et al., 2018) at varying time scales (Murray et al., 2014). Meanwhile, invariance by identity-preserving transformations helps avoid the exponential explosion of PNG numbers. At the core of computation lies the task of novelty detection (e.g., by hippocampus Knight, 1996)—i.e., when should a new PNG be recruited to represent the sensory stimuli?

We note that the definition of novelty is also scale-dependent. Generally speaking, the hierarchical formation of PNGs reflects the mirroring of the internal representation with respect to the nested structures of the sensory stimuli. A stimulus that is locally familiar (e.g., a simple edge) might be globally novel (e.g., a new combination of simple edges). How do simple features get integrated into complex ones is a long-standing open problem called binding (Treisman, 1996) in the literature. We conjecture that two mechanisms play a fundamental role in scale-dependent novelty detection and the related binding problem: (1) sequential firing (generalizing a simple cell) that concludes the selective recognition of a sensory stimulus from a cluttered background, and (2) parallel firing (generalizing a complex cell) (Treisman, 1996) that contributes to the invariant recognition of the object identity regardless of various transformations. Together, they contribute to the formulation of PNGs representing both novel identities and identity-preserving transformations from the local to the global scale.

How do PNGs encode identity-preserving transformations? The temporal stability of sensory stimuli (e.g., the grandmother's face) serves as an implicit supervision signal during object recognition. Environmental uncertainty factors, such as varying poses and positions within a short time span (e.g., 100 m), are assumed to be associated with the same object identity. Therefore, conceptually similar to the spatial invariance achieved by complex cells, we can generalize it to higher-order geometric invariance by a similar max-pooling operation. However, we note that the objective of parallel firing (in contrast to the sequential one) is to simultaneously convey the identity information along with those uncertainty factors. Along this line of reasoning, both the selective recognition of complex patterns (as the superposition of simple ones) and their invariance recognition (through encoding identity-preserving transformations) require an astronomical number of PNGs. We believe that nature has discovered an elegant solution to this problem—i.e., the combinatorial barrier can be overcome by the exponential growth of PNGs from V1 to IT (or over-parameterization). As shown in Izhikevich (2006), the total number of PNGs can be much larger than the number of neurons due to their self-organization by spike timing-dependent plasticity (STDP) (Caporale and Dan, 2008).

In summary, the objective of object recognition at the computational level is to strike a trade-off between selectivity and invariance along the hierarchy. From localized simple patterns to globally integrated features, the nested organization of the physical world is hierarchically mirrored by the internal representation (Rumelhart et al., 1985) (a.k.a. mental model Gentner and Stevens, 2014) of an organism. Such internal representation will serve as the foundation of intelligence for predictive coding. Our generalization of simple and complex cells attempts to unify selectivity and invariance computation by distinguishing between sequential and parallel firing mechanisms. Even though both count on an astronomical number of PNGs as the information carrier, we have found that the evolution of mammalian brains does support such a simple yet elegant solution to object recognition (i.e., recruit more PNGs for selective and invariant computation along the hierarchy). Next, we elaborate on specific algorithms to perform the computations required by object recognition at the algorithmic level.

## 3. Algorithmic level: unsupervised clustering via hierarchical sparse coding

At this level, we break down the computational goal of the previous section into specific processes including data structures and coding algorithms. The unifying theme is to exploit the blessing of dimensionality by generalizing the existing multi-resolution analysis in the Hilbert space (Mallat, 1989) to a high-dimensional topological manifold. A common criticism of increasing dimensionality is concerned with the so-called *curse of dimensionality* (Bellman, 1966) from the perspective of computational complexity. There are several ways to address this problem. First, experimental studies such as Chen et al. (2013) have demonstrated the blessing of dimensionality in face verification applications, which is consistent with biological findings (McNaughton, 2010). Second, recently proposed over-parameterized models (Arora et al., 2018) and direct fit to nature (Hasson et al., 2020) suggest that dimensionality can be a blessing when a large amount of training data is available due to a counterintuitive phenomenon called “concentration of measure” (Ledoux, 2001). Over-parameterization has been shown to be beneficial for both representation learning and few-shot learning (Sun et al., 2021). Third, as dimensionality increases, the existence of many comparable solutions can be exploited by a global optimization algorithm to accelerate the search (assuming any solution works equally well).

### 3.1. Unsupervised clustering on product manifold

As argued by Jean Piaget, the order of grasping mathematical structures in early childhood cognitive development (topology before geometry) is the opposite of what we have learned in school (topology after geometry) (Piaget and Cook, 1952). Therefore, we attempt to construct a biologically plausible object recognition model in a topological space with the least number of assumed mathematical structures before discussing its implementation at the algorithmic level. In this paper, we formalize the biological object recognition as the following unsupervised clustering problem and present algorithmic solution based on sparse coding.

**Problem formulation of biological object recognition**

*Given visual stimuli with a combinatorial number of patterns in varying dimensions, group them into different classes/objects in an unsupervised manner*.

Both subspace and product topologies have their uniqueness in terms of satisfying the characteristic property (Kelley, 2017). The geometric intuition behind our construction of the new hierarchical model is best illustrated by the duality between space partitioning (i.e., subspace topology) and composition (i.e., product topology). Manifold structure under subspace projection has been well studied in the literature (e.g., Johnson—Lindenstrauss lemma Ailon and Chazelle, 2006). By contrast, the other direction (i.e., product topology) has been under-researched. The low-dimensional manifold structure can still be preserved after space composition due to the following lemma.

**Lemma 1: Topological manifold with Cartesian product** (Lee et al., 2009)

*In topology, a topological manifold is a topological space that locally resembles the real* *n-dimensional Euclidean space. Let* *M* *be a topological* *m-manifold and* *N* *be a topological* *n-manifold, then* *M*×*N* *(Cartesian product of* *M* *and* *N) is a topological* (*m*+*n*)*-manifold*.

This lemma explains the blessing of dimensionality in that the manifold structure is easier to discover in a high-dimensional space. Note that manifold learning in a higher-dimensional space requires more training data. For example, the Cartesian product of a horizontal edge and a vertical edge will produce several combinations including “T,” “+,” “⊢,” “⊣, ” and “⊥”. Through combinatorial coding, our selectivity-invariance model stores different patterns like the *k*-d tree (i.e., the centroids of vector quantization or dictionary atoms of sparse coding), but the combination of these basic patterns will be enumerated through product topology to support the direct-fit by approximate neighborhood search to achieve invariant recognition. This combinatorial perspective differs from the HMAX model (Riesenhuber and Poggio, 1999) in which no discrimination was considered for the combination of basic patterns. We argue that the combinatorial coding argument is consistent with the direct-fit model (Hasson et al., 2020) and the theory of PNG (Izhikevich, 2006).

Empirical evidence to support such combinatorial coding arguments is that dynamic and transient synchronization of neural groups self-organizes into functionally coherent assemblies (Singer, 2001). This iteration of self-similar cognitive operations is achieved by the higher-level layers that process the output of the lower-level layers in the same way as these low-level layers process their respective input. To achieve the objective of parallel firing for invariance computation, we consider an extension of the *k*-d tree from fixed-dimension to varying-dimension to exploit the manifold constraint (Lemma 1) as follows.

**Definition: Product manifold tree (PM-Tree)**

*The product manifold tree (pm tree) can be defined as the dual operation of the classic k-d/rp tree. Instead of space partitioning, we recursively merge low-dimensional manifolds in subspaces into higher-dimensional manifolds through product topology*.

How to directly fit the data in a varying-dimensional space to the PM-tree? This problem has been formulated as unsupervised learning in the literature on machine learning (ML) (Barlow, 1989). Unfortunately, all existing work on unsupervised learning and cluster analysis assumes a fixed dimensionality with the input data and focuses on learning a distance metric to discover the local geometry of the manifold (Davis et al., 2007). Our construction of the PM-tree attempts to overcome such a barrier by generalizing *k*-d tree-based clustering with product topology. The performance of the nearest neighbor (NN) search is known to degrade in high-dimensional space, partly due to the surprising behavior of the distance metric as the dimensionality increases (Aggarwal et al., 2001). However, such limitations can be overcome by using the following lemma (note that we do not assume any definition of a distance metric on a topological manifold).

**Lemma 2: Unsupervised clustering on PM-tree**.

*Let* **Z** = **X**×**Y** *be the Cartesian product of two topological manifolds. For a concatenated vector* *z* = [*xy*]*, its neighborhood search can be carried out by taking the intersection of the neighborhoods of* *x*∈**X** *and* *y*∈**Y***, respectively*.

**Sketch of the proof**. It is known that the subspaces and products of the Hausdorff spaces are Hausdorff. One property of product topology is that if $B_{i}$ is a basis for the topology of **X**_*i*_, then the set $\left\{{\text{B}}_{1}\times ...\times {\text{B}}_{n}:{\text{B}}_{i}\in B_{i}\right\}$ is a basis for the product topology on **X**_1_×… × **X**_*n*_ (Lee et al., 2009). The result follows directly from the definition of a neighborhood basis in the topological manifold.

From local to global, such hierarchical construction with simple and complex cell layers allows an organism to directly fit the visual stimuli of varying dimensionality into the hierarchical model by an approximate nearest neighbor (ANN) search in the latent space. Both object recognition and identity-preserving transformations boil down to the search of ANN along the hierarchy. Through recursive space partitioning and composition, our constructed hierarchical model solves the problem of untangling the object representation by directly fitting the stimuli to the PM-tree characterizing the geometry of the neural population (Chung and Abbott, 2021). In the literature, the neural collapse has been observed as a signature of over-parameterized neural networks (Papyan et al., 2020) despite the lack of biological connection. Note that over-parameterized models also need to be parsimonious due to ecological constraints such as non-negativity and energy efficiency (Whittington et al., 2022a). To implement selectivity and invariance computation, we first present a hierarchical extension of convolutional sparse coding (CSC) and then advocate a dynamic-programming (DP)-like approach to object recognition by approximate nearest neighbor (ANN) search.

### 3.2. Hierarchical extension of convolutional sparse coding

It is well-known that sparse coding offers a powerful analysis of the mechanism of V1 (Olshausen and Field, 1996, 1997). Meanwhile, sparse representations have also found promising applications in unsupervised learning, such as K-SVD dictionary learning (Aharon et al., 2006) and non-negative matrix factorization (Lee and Seung, 1999). Unlike predetermined dictionaries (e.g., wavelet Mallat, 1989), data-adaptive dictionary learning such as K-SVD-based sparseland (Aharon et al., 2006) has led to a multilayer formulation of convolutional sparse coding (ML-CSC) (Papyan et al., 2017, 2018), which provided an attractive new theoretical framework for analyzing CNN.

**Multi-layer convolutional sparse coding for selectivity computation**. The new insight brought about by ML-CSC (Papyan et al., 2017, 2018) is to generalize the original sparse coding in a multilayer manner. Specifically, a multilayer convolutional sparse coding (ML-CSC) model can be constructed as follows. Suppose that **X** is the input signal and a set of dictionaries is given by ${\left\{{\text{D}}_{k}\right\}}_{k=1}^{K}$ where **D**_*k*_ denotes the dictionary at the level *k*. Then an ML-CSC model can be written as: **X** = **D**_1_Γ_1_, Γ_1_ = **D**_2_Γ_2_,…, Γ_*K*−1_ = **D**_*K*_Γ_*K*_ where Γ_*i*_ = [*w*_1_, …, *w*_*k*_] denotes the sparse coefficients at the level *i*. Following the convex approximation of ℓ_0_-optimization in Papyan et al. (2017), a layered thresholding algorithm runs recursively as follows.


(1) $$\begin{array}{l} {\text{Γ}}_{k}=P_{\beta_{k}}\left({\text{D}}_{k}^{T}{\text{Γ}}_{k-1}\right),\left(k=1,2,...,K\right) \end{array}$$

where $P_{\beta_{k}}$ is the standard thresholding operator and ${\left\{\beta_{k}\right\}}_{k=1}^{K}$ is the set of thresholds at level *k*.

As shown in Papyan et al. (2018), the ML-CSC model manages to decompose a signal **X**∈*R*^*n*^ into the superposition of multiple dictionaries **D** = **D**_1_**D**_2_…**D**_*K*_ but the concatenation of these atoms, although it is overcomplete, remains in the space of the same dimension *R*^*n*^. Unfortunately, both K-SVD and ML-CSC are still constructed within the Hilbert space without a change of dimensionality, therefore matching our space partitioning abstraction of simple cells. To our knowledge, there is almost no previous work in the open literature that extends the data-adaptive sparse representation to varying dimensions for space composition abstraction of complex cells.

**Figure 2:**
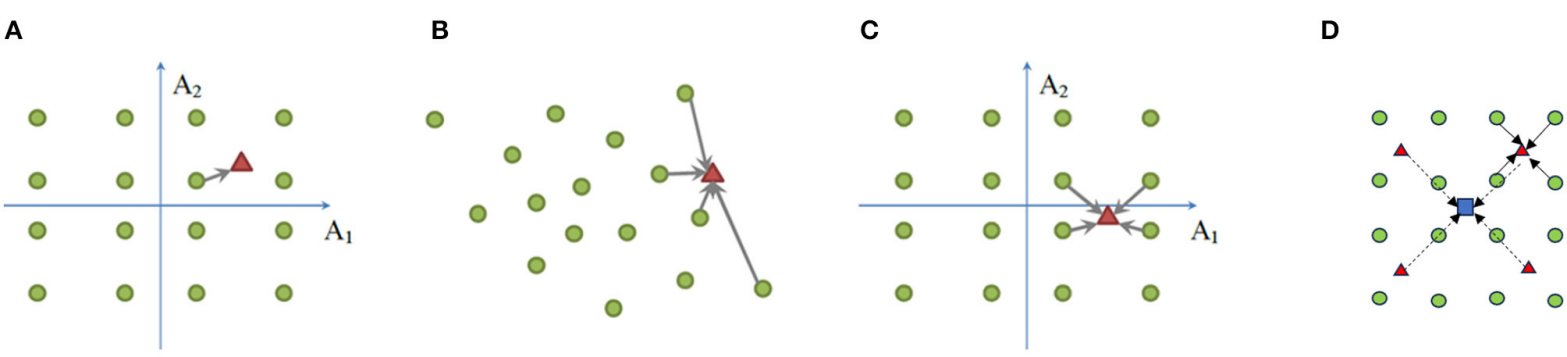
Illustration of hierarchical product sparse coding. **(A)** Product quantization (PQ), **(B)** sparse coding (SC), **(C)** product sparse coding (PSC), and **(D)** hierarchical extension of PSC. **(A–C)** The green circle denotes a codeword, and the red triangle denotes a vector to be encoded; **(D)** the blue square denotes the vector to be encoded at the next level when red triangles become codewords.

Parallel to the product-manifold lemma, we are interested in developing a recursive strategy to decompose a high-dimensional sparse coding problem into the “product” of multiple lower-dimensional ones, which gives the name “product sparse coding” (PSC) (Ge et al., 2014). Let **X** = **D**_*x*_Γ_*x*_ and **Y** = **D**_*y*_Γ_*y*_ denote two dictionary coding schemes with ${\text{D}}_{x},{\text{D}}_{y}\in R^{n\times m},\left(m>n\right)$. Then we start with a coding scheme in the direct-sum or Cartesian product space *R*^2*n*^ by $\left[\begin{array}{c} X\\ Y \end{array}\right]=\left[\begin{array}{cc} D_{x} & 0\\ 0 & D_{y} \end{array}\right]\left[\begin{array}{c} {\text{Γ}}_{x}\\ {\text{Γ}}_{y} \end{array}\right]$and improve the sparsity by basis pursuit algorithm (Chen et al., 2001) using the composite dictionary [**D**_*x, i*_**D**_*y, j*_], (1 ≤ *i, j* ≤ *m*) (total *m*^2^ atoms). Such a process of basis composition via Cartesian product can be recursively applied to obtain sparse bases in higher-dimensional spaces (i.e., *R*^*n*^→*R*^2*n*^→*R*^4*n*^…). A two-level hierarchy of product sparse coding is shown in Figure 2D, which extends previous designs in Figures 2A–C. In summary, the manifold constraint can still be exploited by selectivity computation regardless of the dimensionality. We only need to focus on the objective of achieving invariance next.

**Identity-preserving transformation for invariance computation**. In the hypothesis of cortically local subspace untangling (DiCarlo et al., 2012), parallel efforts from ventral streams are to gradually untangle the object manifold like an assembly line until reaching an untangled object representation at the top level (inferior temporal cortex). At the core of this hypothesis is the algorithm implementing identity-preserving transformation (IPT)—i.e., how to simultaneously convey explicit information about an object's identity as well as its contextual information (e.g., pose, scale, position). If each local subpopulation of neurons encodes contextual information associated with an object, we can cast the objective of disentanglement learning as the following sparse coding problem.


(2) $$\begin{array}{l} \text{X}=\text{D}\Gamma ,\text{D}=\left[{\text{D}}_{1},{\text{D}}_{2},...,{\text{D}}_{K}\right],\Gamma \in {\left\{0,1\right\}}^{K} \end{array}$$

where **D** denotes the exemplars (locally sampled points from the object manifold) of the same object but at varying position, scale, and pose. The sparsity of IPT coefficient vector Γ explicitly reflects the physical constraint in the real world—i.e., any object can only occupy a signal spot in the space of position, scale, and pose. This disentanglement of object identity from other contextual information is a significantly new insight brought about by this work. It is both biologically plausible [e.g., there is no need to rebind the identity with contextual information at a later stage, nullifying the so-called binding problem (Treisman, 1996)] and computationally efficient because the subspaces associated with contextual information are independent of each other [i.e., admitting one-hot coding (He and Chua, 2017)]. The biological plausibility of IPT has been well documented by the study of IT population firing (DiCarlo et al., 2012).

### 3.3. Object recognition via approximate nearest neighbor search

Our hierarchical extension of CSC for selectivity and invariance computation shares a spirit similar to that of dynamic programming in that optimal substructures contribute to the (nearly) optimal solution. Mathematically, ℓ_0_-optimization is an NP-hard problem; but in biology, evolution does not have foresight, and therefore the hierarchical systems generated by evolution might not have a globally optimal structure. What matters more appears to be the nearly decomposability of complex systems (Simon, 1962), which is closely related to the principle of dynamic programming (DP) (Bellman, 1966). Our intuition is that evolution does not need to pursue a globally optimal solution such as nearest neighbor (NN), but be satisfied with an approximate yet flexible solution so that the organism can adapt to the constantly evolving environment. Based on this observation, we next connect object recognition with a DP-like recursive solution to approximate NN (ANN) search.

Instead of ℓ_0_-optimization, we conjecture that ℓ_∞_-optimization [a.k.a., minimax optimization (Hayakawa and Suzuki, 2020)] is a more appropriate framework for analyzing the processing of ventral streams for the following reasons. First, redundancy has been extensively exploited in information theory for reliable communication (Shannon, 1948). The neocortex faces a similar challenge of robustness to errors (e.g., sensory deprivation and lesions), especially for the high-level layers responsible for important decisions related to behavior. In the literature, it has been shown in Lyubarskii and Vershynin (2010); Fuchs (2011) that ℓ_∞_-optimization leads to the so-called spread representation (Fuchs, 2011) where all coefficients are of the same order of magnitude. Such a class of representations is known to robustly withstand errors in their coefficients. Second, the anti-sparse coding scheme based on ℓ_∞_-optimization is known to facilitate the search for ANN (Jégou et al., 2012).

Now we see how both selectivity and invariance computation can be implemented by ANN search. For selectivity computation, we have identity-related dictionaries constructed from the recursive application of Equation (1), which essentially determines the inter-class decision boundary between different objects. For invariance computation, we have identity-excluded dictionaries constructed by Equation (2), which is responsible for shaping the intra-class distribution dictated by IPT. ANN search will simultaneously resolve the uncertainty of both identity(ID)-related and non-ID-related codes in the latent space. Such formulation can also be interpreted as multi-task learning (Caruana, 1997) where object recognition and uncertainty modeling (e.g., pose estimation) mutually serve as the tool of regularization. As the field-of-view (dimensionality of visual stimuli) increases, the sparsity of spread representations also increases, contributing to the acceleration of ANN search (Cherian, 2014). More importantly, simultaneously learning multiple tasks can facilitate the exploitation of the similarity between selectivity and invariance computation to improve the joint sparsity (Calandriello et al., 2014).

The above interesting connection implies that it is easier to construct a DP-like solution by decomposing the high-dimensional ANN search problem into several subproblems in projected subspaces (Jegou et al., 2010). Combining anti-sparse coding with hierarchical CSC, we can envision a DP-like recursive solution to object recognition via ANN search in high-dimensional space. Thanks to the construction of hierarchical CSC, we can recursively construct a dictionary in a high-dimensional space from the direct product of dictionaries in low-dimensional spaces [similar to product quantization (Jegou et al., 2010)]. It follows that an anti-sparse coding scheme in high-dimensional space can be obtained by decomposing it into subproblems in low-dimensional spaces (conceptually similar to the principle of dynamic programming). It should be noted that space partitioning, such as the *k*-d tree and the random projection forest (Yan et al., 2019) also supports the NN/ANN search (Friedman et al., 1977). Therefore, it is plausible that *approximate neighborhood search* strategy without the involvement of a distance metric but supported by the selectivity and invariance computation could serve as a common currency to unify the top-down and bottom-up processing mechanisms in ventral stream processing.

## 4. Implementational level: two possible neural network implementations

In this paper, we present two experimental ideas for testing hierarchical selectivity-invariance algorithms by two network implementations: asymmetric sparse autoencoder (Ng et al., 2011) and divergent spiking neural networks (SNN) (Eguchi et al., 2018). It is conjectured that these network architectures might have higher brain hierarchy scores (Nonaka et al., 2021) than existing deep neural networks.

### 4.1. Asymmetric sparse autoencoder

**Figure 3:**
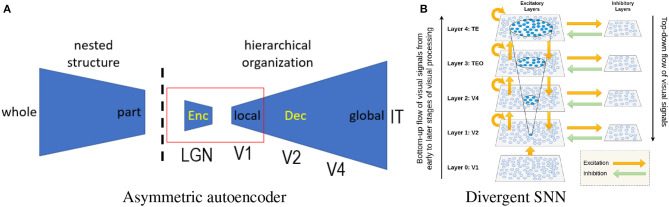
**(A)** Architecture of an asymmetrical autoencoder (the dashed line marks the boundary between environment and organism; the red box corresponds to the standard symmetric autoencoder). **(B)** Divergent SNN (there are more cells and synapses at higher levels than at lower levels).

The autoencoder represents a popular network architecture for unsupervised learning. A straightforward application of the sparse coding principle to the autoencoder is possible (Ng et al., 2011). The hierarchical extension of the CSC inspires us to consider the design of an asymmetric autoencoder, as shown in Figure 3A. Its asymmetric design is also biologically inspired by Barlow (2001) redundancy exploitation hypothesis. The hierarchical organization of the neocortex is believed to reflect the nested structure of the physical world, indicating that the decoder (responsible for the reconstruction of internal representations) plays a more important role than the encoder. More specifically, we can implement a prototype by combining an over-parameterized autoencoder (Radhakrishnan et al., 2019) with manifold-based novelty detection. In the literature, autoencoder-based representation learning (Tschannen et al., 2018) is known to be capable of learning disentangled and hierarchical representations. Instead of storing training samples as attractors (Radhakrishnan et al., 2019), we envision that the latent space of the autoencoder includes not only identity information but also IPT-related information (e.g., latent codes representing the pose, position, scale, and context of an object).

The new insight brought about by our asymmetric design is the increasing dimensionality of the latent space (e.g., from V2 to V4 and IT). Unlike VQ-VAE (Van Den Oord et al., 2017), we do not constrain the latent capacity but allow the space of latent codes to have even higher dimensionality than the input data. Our intuition is that in the presence of a new category/object, the decoder will be further expanded to accommodate the novelty class like in the hippocampus (i.e., consolidation of new memory). Such unsupervised and continual learning can lead to a monotonically increased memory capacity for associative memory implemented by the asymmetric autoencoder. Unlike (Radhakrishnan et al., 2019) treating sequence encoding as composition maps and limit cycles, we argue that a biologically more plausible mechanism for memory storage is based on the rich interaction between sensory and motor systems. From the selectivity-invariance perspective, motion dictates the regime within which the organism achieves invariance to the composition of geometric transformations. The rich interaction between sensory and motor systems contributes to the formulation of reconstruction problems on multiple scales from parts to the whole (Hinton, 2021) as an unsupervised mechanism for learning invariant representations. When the sensory motion goes out of the normal range (e.g., rotating a book continuously), the asymmetric sparse autoencoder is supposed to fail to recognize the object (i.e., it will be treated as a novelty), which is a testable prediction.

To implement hierarchical PSC on asymmetric autoencoder, we can extend the existing supervised translation-invariant sparse coding (Yang et al., 2010) in the following aspects. First, instead of sparse subspace modeling, our selectivity-invariance perspective allows us to pursue a sparse product-space modeling of visual stimuli. Locally image descriptors from two classes can be made most separable by sparse coding; globally, such linear separability is generalized into an ensemble of linearly separable functions in a higher dimensional space after Cartesian product. Biologically, the population of IT neurons corresponds to this collection of linearly separable functions (e.g., the binary facial attributes of a face image). Second, instead of translation-invariant sparse coding, asymmetric autoencoder allows us to achieve invariance to more generic geometric transformations (e.g., pose, scale, rotation). One concrete idea of implementation is to extend dilated/atrous convolutions into a rotated convolution (Pu et al., 2023) or even deformable convolutions (Dai et al., 2017), which can be interpreted as identity-preserving transformations (DiCarlo et al., 2012).

### 4.2. Divergent spiking neural networks

In previous work (Izhikevich, 2006), polychronization was conceived as a basic mechanism for computing with spikes. It is built upon Hebb's postulate but extends it by relaxing the synchronous firing into polychronous time-locked patterns. Therefore, the group of neurons that are spontaneously organized by the fundamental process of spike-timing-dependent plasticity (STDP) is called Polychronous Neuronal Group (PNG). The mechanism of polychronization has recently been studied in Eguchi et al. (2018); Isbister et al. (2018) as a plausible solution to the problem of feature binding. Using a spiking neural network (SNN), input training images (sensory stimuli) can be mapped to a hierarchy of PNGs by the emergence of polychronization across hierarchical timescales (Murray et al., 2014).

Unlike previous studies (Eguchi et al., 2018; Isbister et al., 2018), our over-parameterized selectivity-invariance model can be implemented on SNN with a divergent or expansive architecture (Babadi and Sompolinsky, 2014), as shown in Figure 3B. Such a divergent architecture directly matches our intuition of generalizing complex cells by space composition. It is easy to see that the number of PNGs can grow exponentially as the number of neurons increases. Even though theoretically it is difficult to exactly count the total number of PNGs, this number is estimated to be much larger than the total number of neurons. Therefore, the memory capacity of PNG is exponential, surpassing the capacity of any known models of associative memory [e.g., Hopfield network (Hopfield, 1982)]. What is less known is how such an astronomical number of PNGs supports neural computation, including sensory perception and cognitive functions. The new insight brought by PNG theory is that the occurrence of polychronization signifies something important or meaningful—e.g., recognized objects, bound features, or shifted attention.

The hierarchical formation of PNGs reflects the mirroring of the internal representation with respect to the nested structures of the sensory stimuli. The causality of STDP dictates the compositional relationship between low-level and high-level visual features. The superposition requires an astronomical number of PNGs, but this barrier can be overcome by the exponential growth of PNGs from V1 to IT. The biological constraint such as processing latency, helps us better appreciate the potential (i.e., unprecedented memory capacity with realistic delay) of Hebbian plasticity. One way to experiment with our divergent SNN is to focus on its memory capacity—e.g., recently developed variable binding implementation (Frady et al., 2021) can be considered in combination with hierarchical timescales (Murray et al., 2014). A testable prediction is that our divergent SNN can recognize more patterns than the original SNN with a convergent architecture (Eguchi et al., 2018).

The training of the proposed SNN can be implemented by a recently developed algorithm named backpropagated neighborhood aggregation (BP-NA) (Yang et al., 2021) or evolutionary structure learning (ESL) (Shen et al., 2023). The key insight behind BP-NA is closely related to the approximate NN search in our previous section. Instead of attempting to differentiate the non-differentiable spiking activation functions, BP-NA computes the aggregated gradient from a properly defined neighborhood. It is worth noting that the latest advance in neuromorphic computing [e.g., Intel's Pohoiki Springs (Frady et al., 2020)] has demonstrated a scalable approximate-NN (ANN) algorithm for searching large databases. We can further accelerate the ANN search by decomposing a high-dimensional ANN search into several subproblems in the projected subspaces [e.g., DP-based optimized product quantization (Cai et al., 2016)]. It is possible to convert the asymmetric autoencoder into an SNN-based implementation based on recent works (Xu et al., 2021, 2022, 2023a,b).

## 5. Application to face identity coding and thatcher illusion

### 5.1. Face identity coding

In face representation, there exist two competing hypotheses about the coding of facial identities. In exemplar-based coding (a.k.a., grandmother cell hypothesis), a subset of neurons in the medial temporal lobe (MTL) fire selectively to strikingly different images of an individual (e.g., Jennifer Aniston), landmarks, or objects and in some cases even by letter strings with their names (Quiroga et al., 2005). In axis-based coding (a.k.a., feature-based coding), face-selective neurons display flat tuning along dimensions orthogonal to the axis being coded; single neurons in the inferotemporal (IT) cortex project input faces, represented as vectors in face space, onto specific axes. Apparently, from the IT cortex to the MTL, we observe a transformation of complex visual percepts into long-term and more abstract memories. Our selectivity-invariance model along the hierarchy offers a possible implementation of such a transformation—from V1 to IT, selectivity and invariance computation jointly contribute to the encoding of facial identities into a latent feature space; from IT to hippocampus, the identity information along with other latent features (e.g., age, race, gender, social trait) and possibly other modalities (e.g., speech) will be passed together to form an abstract concept such as one's grandmother. One can design a perturbation experiment to test how much distortion is needed to change the perception of a grandmother to a different identity.

### 5.2. Thatcher illusion

**Figure 4:**
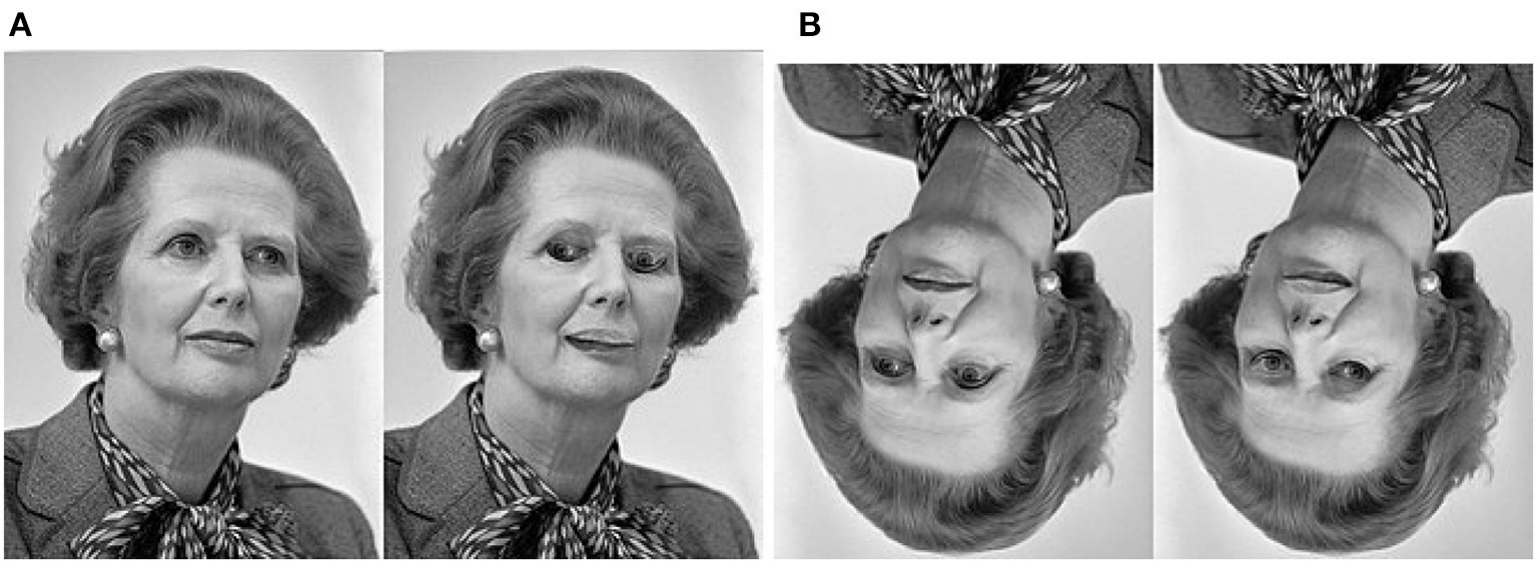
Thatcher effect. It is much easier to detect inverted eyes from **(A)** an upright face than from **(B)** an upside-down face. This is due to the top-down prediction made by the PNG at the IT layer. Only in the upright face can the discrepancy between predicted and actual orientations of facial landmarks be detected as a novelty. In the upside-down face, we can only tell the global orientation because there is no binding for local landmarks and global impressions (Thatcher effect.jpg, 2020).

Thatcher illusion (refer to Figure 4) occurs when it is difficult to detect local feature changes in an upside-down face, despite identical changes being obvious in an upright face (Thompson, 1980). To explain this illusion, we note that (1) this illusion can be viewed as a special case of anomaly detection where local perception has a conflict with global one; and (2) face is unique due to its significant role in our social communication. Through evolution and development, humans have developed specific processes for recognizing holistic faces as well as salient facial landmarks (i.e., eyes, nose, and mouth). In the situation of upright faces, inverted eyes, and mouths become anomalies because their positions and relationships as characterized by the one-hot encoded vector Γ in Equation (2) have changed. By contrast, in the case of an inverted face, the holistic impression (the novelty of upside-down) is prevalent, but our brain does not have the corresponding prediction about the local orientations and spatial relationships of facial landmarks. Each landmark is processed independently, making it more difficult to tell whether it is inverted or not. One can make a testable prediction about the middle ground between Figures 4A, B—as the image pair is rotated clockwise from 0° to 180°, there must exist a phase transition period for the activation of the Thatcher effect.

### 5.3. Kanisza's illusion

**Figure 5:**
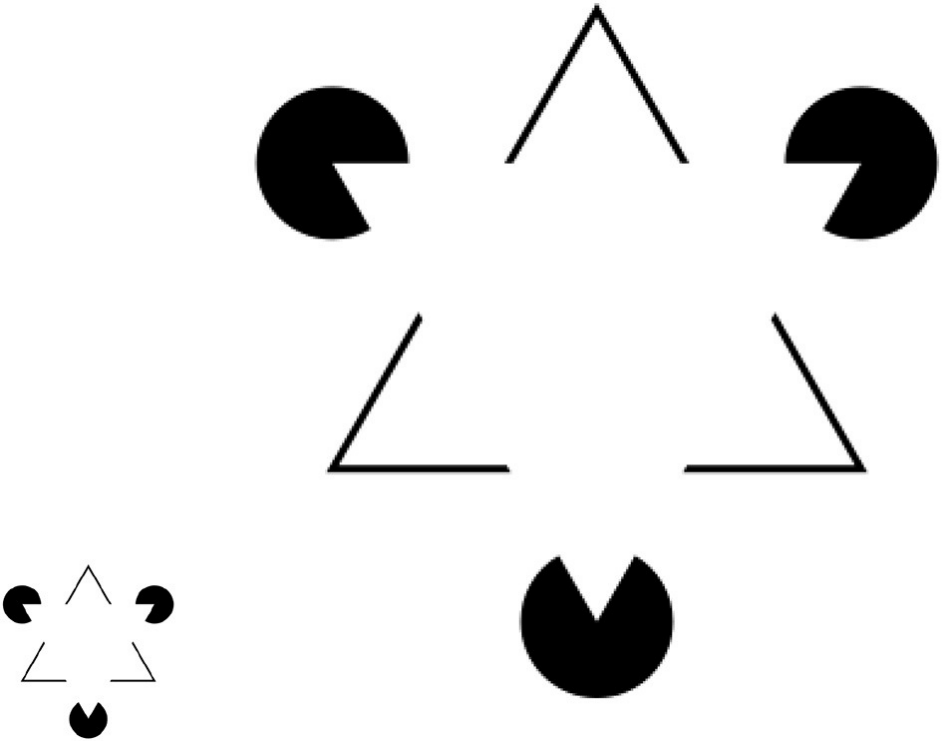
The illusory triangle in the original Kanizsa's triangle **(Left)** disappears as the image scales up/zooms in **(Right)** due to the increased distance among three Pacman objects. This example demonstrates the relativity of perceptual organization.

. Kanisza's illusion (Von der Heydt et al., 1984) is a classical example of illustrating an illusory contour (e.g., the impression of seeing a white triangle on top of the black triangle in Figure 5). What has been less studied is the *perturbed* version of this illusion—e.g., as one increases the size of the black triangle and the distance between three Pacmans by zoom-in (the displayed ratio is 400%), it will become more and more difficult to perceive the illusory “white triangle” at the center. Such experimental findings cannot be explained by Gestalt theory because there is no prediction of the critical boundary condition for the perceptual organization to fall apart. Our selectivity-invariance computation principle can predict that the threshold for scale invariance is determined by the size of the fovea centralis. When the distance is above this threshold, there is no previous experience (visual stimulus) to evoke the formation of PNG supporting the perceptual grouping of white triangles. This is another concrete evidence supporting the relevance of optical illusion to previous experience.

## 6. Conclusion

This paper advocates a selectivity-invariance model approach to understanding ventral stream processing at computational, algorithmic, and implementation levels. From the point of view of blessing dimensionality, we revisit the model of simple and complex cells and generalize them into sequential and parallel firing of polychronization neural groups. Through this new perspective of selectivity-invariance computation, we can extend the existing *k*-d tree into the PM-tree to construct a hierarchical sparse coding model. Inspired by the close relationship between object recognition and approximate nearest-neighbor search, we recast the role played by object identity selectivity and identity-preserving transformations from the perspective of manifold untangling. Two possible network implementations and potential applications in the coding of facial identity and the perception of visual illusion are briefly discussed.

## Data availability statement

The original contributions presented in the study are included in the article/supplementary material, further inquiries can be directed to the corresponding author.

## Author contributions

XL: Conceptualization, Writing—original draft, Writing—review and editing. SW: Conceptualization, Funding acquisition, Writing—review and editing.
